# Physiologically vulnerable or resilient? Tropical birds, global warming, and redistributions

**DOI:** 10.1002/ece3.9985

**Published:** 2023-04-18

**Authors:** Otto Monge, Ivan Maggini, Christian H. Schulze, Stefan Dullinger, Leonida Fusani

**Affiliations:** ^1^ Vienna Doctoral School of Ecology and Evolution University of Vienna Djerassiplatz 1 1030 Vienna Austria; ^2^ Konrad‐Lorenz Institute of Ethology University of Veterinary Medicine Savoyenstrasse 1a 1160 Vienna Austria; ^3^ Department of Botany and Biodiversity Research University of Vienna Rennweg 14 1030 Vienna Austria; ^4^ Department of Behavioural and Cognitive Biology University of Vienna Althanstrasse 14 1090 Vienna Austria

**Keywords:** anthropocene, bird conservation, ecophysiology, thermal stress

## Abstract

Tropical species are considered to be more threatened by climate change than those of other world regions. This increased sensitivity to warming is thought to stem from the assumptions of low physiological capacity to withstand temperature fluctuations and already living near their limits of heat tolerance under current climatic conditions. For birds, despite thorough documentation of community‐level rearrangements, such as biotic attrition and elevational shifts, there is no consistent evidence of direct physiological sensitivity to warming. In this review, we provide an integrative outlook into the physiological response of tropical birds to thermal variation and their capacity to cope with warming. In short, evidence from the literature suggests that the assumed physiological sensitivity to warming attributed to tropical biotas does not seem to be a fundamental characteristic of tropical birds. Tropical birds do possess the physiological capacities to deal with fluctuating temperatures, including high‐elevation species, and are prepared to withstand elevated levels of heat, even those living in hot and arid environments. However, there are still many unaddressed points that hinder a more complete understanding of the response of tropical birds to warming, such as cooling capacities when exposed to combined gradients of heat and humidity, the response of montane species to heat, and thermoregulation under increased levels of microclimatic stress in disturbed ecosystems. Further research into how populations and species from different ecological contexts handle warming will increase our understanding of current and future community rearrangements in tropical birds.

## Graphical abstract

Emprirical data from the literature show that tropical birds are not characterized by narrow thermal tolerance and may not be physiologically threatened by global warming in the short term.

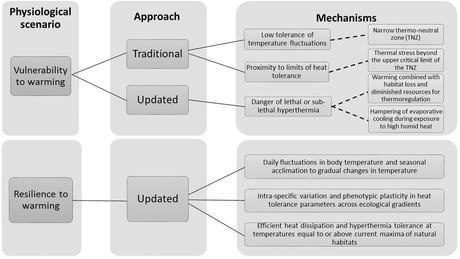

## 1 INTRODUCTION

The effects of anthropogenic climate change can be particularly pervasive in tropical ecosystems (Foden et al., 2013; Laurance et al., 2011). For example, recent predictions suggest that end‐of‐century temperatures could surpass the realized thermal limits of proportionally more organisms in the Tropics than at higher latitudes (Trisos et al., 2020). In consequence, redistributions of tropical communities are expected to occur more frequently (Freeman et al., 2021). Distributions would shift along elevational gradients, with species at the mountain tops being particularly disadvantaged given the spatial limitations for expanding further upwards (Freeman et al., 2018; Marris, 2007). In turn, the lowest elevations would suffer from biotic attrition because upward shifts and local extinctions of their biotas may not be compensated by species moving in from still warmer areas (Colwell et al., 2008). Following these rearrangements, turnovers benefitting warm‐adapted species would lead to the thermophilization of communities (Fadrique et al., 2018).

It is commonly assumed that the main driver of the observed distributional rearrangements in tropical biotic communities is thermal sensitivity (Khaliq et al., 2014; Laurance et al., 2011) because many organisms are thought to live near their thermal tolerance limits, beyond which survival is compromised, already under current climatic conditions (Trisos et al., 2020) and to possess low tolerance to temperature variation (Tewksbury et al., 2008). These assumptions have been derived from the narrow distribution ranges (e.g., elevational) of many tropical species, which apparently suggest narrow thermal niches and hence high thermal sensitivity (Colwell et al., 2008; Laurance et al., 2011). Yet, sound empirical evidence for these assumptions coming from physiological studies is surprisingly limited. In fact, most studies that measured thermal tolerance in relation to the warming expected over the next decades focused on tropical ectotherms so far [e.g., *Anolis* lizards (Logan et al., 2014); ants (Tizón et al., 2014); littoral snails (Marshall et al., 2015); amphibians (von May et al., 2019)].

For tropical endotherms, there is a large knowledge gap on whether physiological vulnerability to warming underlies distributional rearrangements. In the case of birds, population responses to climate change have been well documented. Biotic attrition and abundance declines have been related to increases in maximum temperature and rainfall alterations (Blake & Loiselle, 2015; Curtis et al., 2021; Tsai et al., 2015). Elevational shifts (Forero‐Medina et al., 2011; Freeman et al., 2018; Freeman & Class‐Freeman, 2014; Neate‐Clegg et al., 2021; Peh, 2007), and thermophilization of montane (Neate‐Clegg et al., 2021; Williams & de la Fuente, 2021) and lowland bird communities (Curtis et al., 2021) have also been observed. Accordingly, many authors have underscored the possibility that a diminished capacity to handle temperature variation in tropical birds may be behind population‐level responses (Curtis et al., 2021; Huey et al., 2012; Jirinec, Elizondo, et al., 2022; Khaliq et al., 2014). However, to date, there is no empirical evidence linking these rearrangements to direct physiological sensitivity to warming.

In stark contrast, it is possible that the true thermal niches of at least lowland birds are actually wider than currently realized ones because warmer conditions could be managed but do not currently occur across species ranges (Burner et al., 2019; Freeman & Beehler, 2018; Shoo et al., 2005). This may explain why lowland birds have retained their distributions through decades of warming in an undisturbed Andean forest (Freeman et al., 2018). This possibility might also extend to montane communities that have not experienced changes in elevation limits or abundance (Campos‐Cerqueira et al., 2017; Rosselli et al., 2017), and in which warm‐ and cold‐adapted birds have increased in abundance (Dulle et al., 2016).

Given the void of knowledge on the physiological response of tropical birds to global warming, this aspect should be first addressed before attempting to relate distributional rearrangements to thermal sensitivity (Cahill et al., 2013). In this paper, we conducted a review of literature on thermoregulation in tropical birds when exposed to thermal variation, specifically their response to heat. Our approach consisted of an integrative or synthetic review (Sayer, 2018; Torraco, 2005) for which we performed a search of literature in online databases (Supplementary Material). We focused our synthesis on the circumstances under which tropical birds are vulnerable to warm conditions. We tied together the empirical data from the retrieved studies with the physiological processes that can confer vulnerability or resilience to answer the following questions: are tropical birds characterized by narrow thermal tolerances? Are they currently living close to their thermal tolerance limits? And, consequently, are they particularly vulnerable to warming from a physiological standpoint? We reinforced our analysis by exploring how microclimatic alterations, such as the ones driven by land‐use change, and humidity influence physiological vulnerability. Finally, we identified knowledge gaps and suggested directions for future research that can guide comprehensive analyses of tropical bird vulnerability to the effects of global warming.

## 2 CLIMATE CHANGE AND AVIAN THERMOREGULATION IN THE TROPICS

Recent studies show that the Tropics are warming more and experiencing more extreme heat events than other world regions (Zeng et al., 2021). If a ~5°C warmer future is met, as in worst‐case scenario, by 2100 some tropical regions could have as much as 120 heat‐wave days per season (Perkins‐Kirkpatrick & Gibson, 2017). Hot days in the Tropics are becoming hotter because they are also dry days (Byrne, 2021). Increases in drought stress are expected to occur in many tropical regions due to shifts in the wind patterns that determine the rainfall seasonality across the Tropics (Mamalakis et al., 2021). Moreover, long‐term drying trends have caused large‐scale reductions in terrestrial water storage (Zhou et al., 2014). On the other hand, the frequency of combined events of dangerously high heat following major tropical cyclones is projected to increase from currently three events per 30‐year period to potentially occurring annually, if temperatures rise by up to 4°C in some tropical regions (Matthews et al., 2019).

**FIGURE 1:**
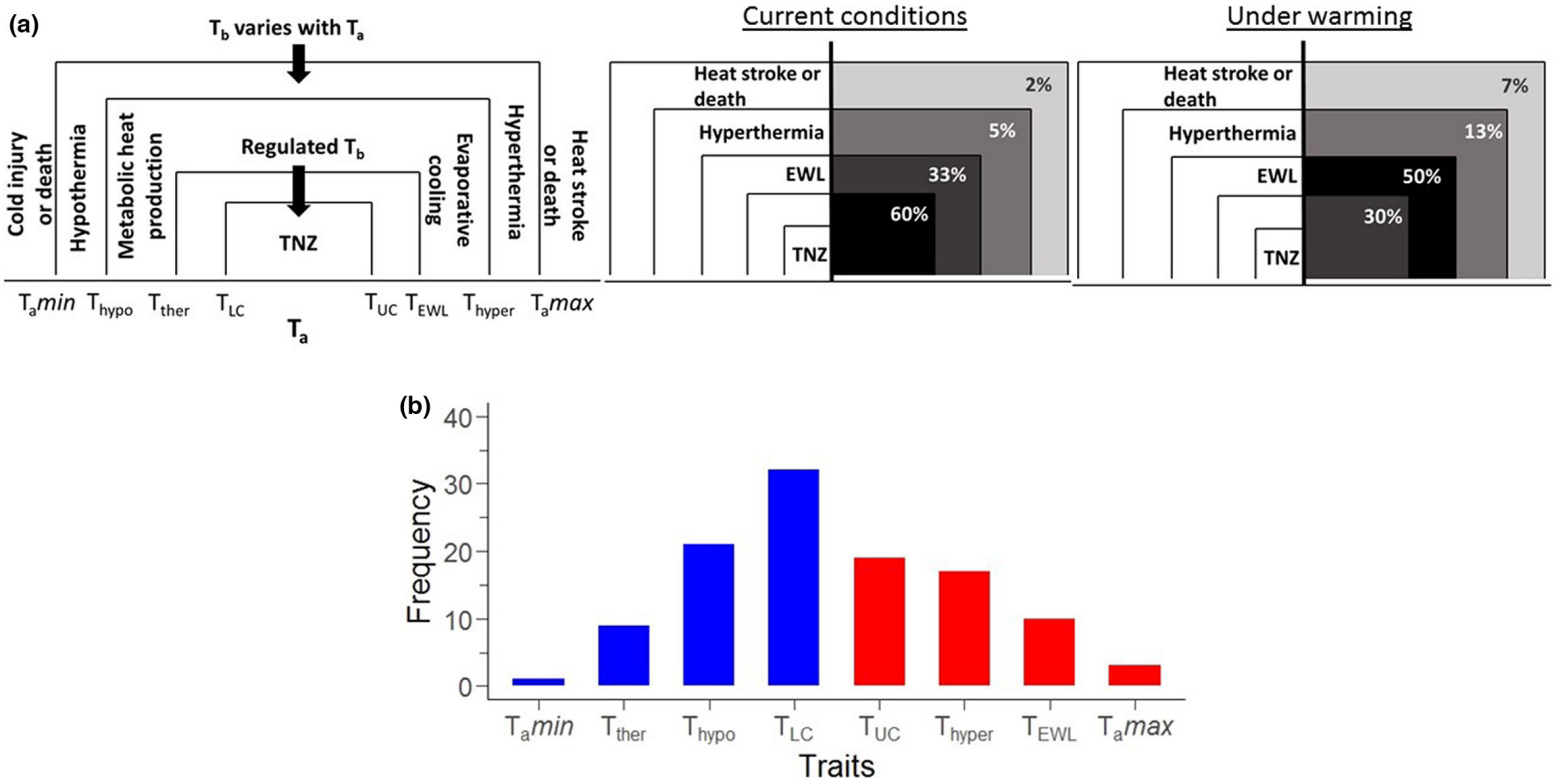
Avian physiological response to thermal variation. (a) Body temperature (**T**_**b**_) is regulated within a range of ambient temperatures (**T**_**a**_), outside of which T_b_ varies with T_a_. Hyperthermia develops when the heat load is not sufficiently dissipated and death may follow thereafter if exposure is prolonged and intense. As a hypothetical example, under current conditions, a tropical bird species living in a hot environment may experience seasonal or yearly dangerous T_a_ for a small proportion of the time (lighter shadings); however, exposure may increase under warming. (b) Frequency in which physiological traits of tropical birds related to the tolerance of cold (blue bars) and hot (red bars) T_a_ appear in studies (*n* = 47 articles, Supplementary Table S1). **T**_**LC**_ and **T**_**UC**_ = lower and upper critical limits of the thermo‐neutral zone (TNZ), **T**_**hypo**_ and **T**_**hyper**_ = T_a_ in which hypo‐ and hyperthermia develop; **T**_**ther**_ = T_a_ that triggers a thermogenesis response to cold (i.e., metabolic heat production); **T**_**EWL**_ = T_a_ that forces a sharp increase in the rate of evaporative water loss (EWL); **T**_**a**_ ***min*** and **T**_**a**_ ***max* =** minimum and maximum tolerable T_a_.

In order to cope with heat stress, physiological mechanisms are set in motion in birds to prevent negative effects to fitness (Figure 1a; Angilletta et al., 2010). In particular, body temperature (T_b_) is regulated when ambient temperatures (T_a_) increase. For this, heat loads produced by endogenous metabolic activity and those absorbed from the environment are dissipated by evaporating water (evaporative water loss, EWL) through the respiratory tract, enhanced by panting in many species, and through the skin (Weathers, 1981). However, at increasingly high T_a_, T_b_ might start to rise uncontrollably and push the bird into heat stress, surpassing the birds' heat tolerance limits (Cabello‐Vergel et al., 2022; Pollock et al., 2021). Reaching the maximum tolerable T_a_ (T_a_ *max*) would then prove fatal (Kendeigh, 1969). More important than the mere exposure is the intensity and duration of the exposure that can determine the heat tolerance limits and ultimately the probability of survival (Rezende et al., 2014). Furthermore, since most birds cannot produce enough water from metabolism to replace losses, several mechanisms can be additionally triggered to conserve water and avoid dehydration (Dawson, 1982). Facultative hyperthermia allows T_b_ to slightly surpass T_a_, thereby creating a thermal gradient in which heat dissipates passively from the body to the surroundings (McKechnie & Wolf, 2019), reducing the need for evaporative cooling (Gerson et al., 2019). Birds may also benefit from maintaining low levels of metabolic activity to avoid generating excess internal heat. Commonly, the rate of metabolic expenditure is measured experimentally when it is at a minimum (basal metabolic rate, BMR) across a given range of T_a_. This range (thermo‐neutral zone, TNZ) is limited by lower and upper critical T_a_ (T_LC_ and T_UC_, respectively) and may vary, along with BMR, across ecological contexts, even among populations of the same species (Castro et al., 1985; Maldonado et al., 2012; McNab, 2013; Tieleman et al., 2002).

Traditionally, the TNZ has been regarded as ecologically important for thermal stress because an increased amount of metabolic energy is invested into maintaining constant T_b_ when T_a_ surpasses its limits (Fristoe et al., 2015; Scholander et al., 1950). In consequence, it has been stated that as long as T_a_ remains within the TNZ, tropical birds are in a thermoregulatory “safe zone,” but when warming T_a_ deviates beyond the T_UC_, survival is threatened or fitness reduced (Khaliq et al., 2014). Despite that the relevance of relying on the TNZ as a measure of thermal tolerance in endotherms has been disputed (e.g., Cabello‐Vergel et al., 2022; Mitchell et al., 2018), it remains a prominent feature in avian physiological studies. In fact, our review of literature evidenced a tendency toward estimating the TNZ and its limits with less attention given to quantifying EWL or T_a_ *max* in tropical birds (Figure 1b). However, assessing the possible consequences of climate‐change driven increases in T_a_ in relation to the TNZ alone may not be the best approach, because many endotherms regulate their T_b_ outside the TNZ through physiological and behavioral strategies and may actually live at T_a_ above their T_UC_ (Freeman et al., 2020; Mitchell et al., 2018). For better insights into possible effects of climate change on tropical bird thermoregulation, it is hence reasonable to jointly examine the variation in the rate of metabolism along with the physiological mechanisms for heat tolerance.

Worldwide, birds from hot and water‐limited environments have been studied in depth because of their obvious state of risk from warming, but virtually all studies come from sub‐tropical deserts. Thus, we scarcely know how tropical birds, and especially rainforest and montane species, deal with heat stress. For instance, what is the variation in the heat tolerance limits and T_a_ *max* of individuals, populations, and species (Boyles et al., 2011; Cabello‐Vergel et al., 2022; Pollock et al., 2021)? Thermal environments that demand a higher evaporative cooling effort may not be limiting to birds if water lost to EWL is replaced, thereby safely maintaining an efficient cooling capacity. Regular drinking is in fact vital to endure T_a_ approaching T_a_ *max* (Czenze et al., 2020; Freeman et al., 2020). On the other hand, with limited access to water, survival can be compromised with sustained exposure even to nonimmediately lethal T_a_ (Mitchell et al., 2018). Subtropical birds who start panting at a relatively low T_a_ are more vulnerable to warming (Pattinson et al., 2020) because prolonged panting can result in dehydration from EWL and also interfere with efficient food and water consumption (Du Plessis et al., 2012; Smit et al., 2016). Moreover, some species may perish if they are unable to withstand severe hyperthermia, even if specialized in conserving body water (Czenze et al., 2020). Thus, in hot, arid subtropical environments, and most likely elsewhere, there will be variation in the degree of vulnerability to warming among bird species depending on their capacity to fulfill cooling requirements (Riddell et al., 2019).

## 3 PHYSIOLOGICAL FEATURES OF TROPICAL BIRDS AND THEIR RELATION WITH VULNERABILITY TO CLIMATE WARMING

Tropical birds would become physiologically vulnerable to warming if unable to efficiently cool down the body when facing extremely high temperatures. The limits of thermal tolerance could be subsequently surpassed and local extinctions may follow. These dynamics promoted the collapse of a subtropical avian desert community (Riddell et al., 2019), but no cases have been reported so far in the wet Tropics. In addition, warming‐induced heat stress within native distributions might lead organisms to redistribute into areas where temperatures match preferred values, chasing their thermal niche when rising temperatures exceed their narrow tolerance of temperature variation (Colwell et al., 2008). However, to date such a direct physiological trigger of distributional changes has not been documented in tropical birds anywhere. In this section, we examine whether tropical birds are actually characterized by a low capacity to handle T_a_ fluctuations and live close to their limits of thermal tolerance under current conditions. We additionally discuss the influence of air humidity and variation in micro‐habitat conditions in the context of vulnerability to warming.

### 3.1 Thermal tolerance capacity

The prevalent notion in the literature is that tropical endotherms possess a narrow TNZ because they inhabit mostly climatically stable habitats and, as a result, they are physiologically vulnerable to temperature variation (Scholander et al., 1950; Sheldon et al., 2018; Stratford & Robinson, 2005). Under this view, many tropical birds are restricted to habitats where T_a_ fluctuates within a very narrow range—presumably the TNZ. When facing fluctuations in T_a_ outside the TNZ limits, birds would thence become thermally stressed. For instance, one tropical montane species was deemed intolerant to T_a_ above a T_UC_ of merely 31°C (Weathers & van Riper, 1982).

However, contrary to the assumption of narrow thermal niches, a growing body of literature shows that the thermal tolerance capacity of many tropical birds is broader than commonly thought (Freeman et al., 2018; Pollock et al., 2021). Experimental measurements evidence a highly variable thermo‐tolerance response to temperature gradients, including T_b_ fluctuating or remaining almost constant within or above the TNZ (Table 1). In fact, some species experience natural daily T_b_ rhythms in amplitudes of ≥10°C (Bartholomew et al., 1983; Cheke, 1970; Morrison, 1962; Schuchmann & Schmidt‐Marloh, 1979a), and, contrary to common belief, the trend for many ecologically diverse tropical birds is to possess broad TNZs of ≥10°C. Thus, it seems unlikely that a narrow tolerance to thermal variation is a fundamental characteristic of tropical birds and that species are restricted to a specific thermal context in consequence.

**TABLE 1:** Reported response of tropical birds exposed to experimental thermal gradients in a representative selection of thermo‐tolerance studies (see Supplementary Material).

Species	Habitat	Thermal gradient (°C)	TNZ range (°C)	T_b_ range (°C)	Reference
*Podargus ocellatus*	Lowland rainforest	5–47	30–40	36–43	Lasiewski et al. (1970)
*Lonchura fuscans*	Lowland open areas	17–44	30–39	38–44	Weathers (1977)
*Bolborhynchus lineola*	Montane humid forest	4–36	28–30	40–42	Bucher (1981)
*Geophaps plumifera*	Lowland arid	−10–51	35–45	41–44	Withers and Williams (1990)
*Amadina fasciata*	Lowland open areas	19–42	31–38	43–44	Marschall and Prinzinger (1991)
*Sporophila corvina*	Lowland rainforest	14–46	29–39	39–47	Weathers (1997)
*Coereba flaveola*	Lowland rainforest	15–40	25–35	35–45	Merola‐Zwartjes (1998)
*Saltator orenocensis*	Lowland dry forest	13–34	28 – ≥34	35–40	Bosque et al. (1999)
*Todus mexicanus*	Lowland rainforest/xeric	15–40	29–≥35	28–43	Merola‐Zwartjes and Ligon (2000)
*Eurillas virens*	Lowland rainforest/xeric	10–35	22–≥35	38–41	Seavy and McNab (2007)
*Hylophylax naevioides*	Lowland rainforest	14–36	30–34	35–42	Steiger et al. (2009)
*Cyanerpes cyaneus*	Lowland rainforest	15–35	25–35	40–41	Mata (2010)

*Note*: All temperature values were rounded to the upper unit. In some cases, an approximate value for the upper critical limit of the thermo‐neutral zone (TNZ) was given. Body temperature (T_b_) range represent minima and maxima during experiments.

### 3.2 Proximity to thermal tolerance limits

If tropical birds live close to their limits of thermal tolerance, their vulnerability would drastically increase when facing warming. For instance, decade‐long trends in extended and warmer dry seasons have been associated with reductions in survival, recruitment, and population growth rates in Central American birds, including one species adapted to lowland dry forests (Brawn et al., 2017; Woodworth et al., 2018). However, without measurements of thermal tolerance, it is unknown whether the observed patterns arise from thermal stress.

**FIGURE 2:**
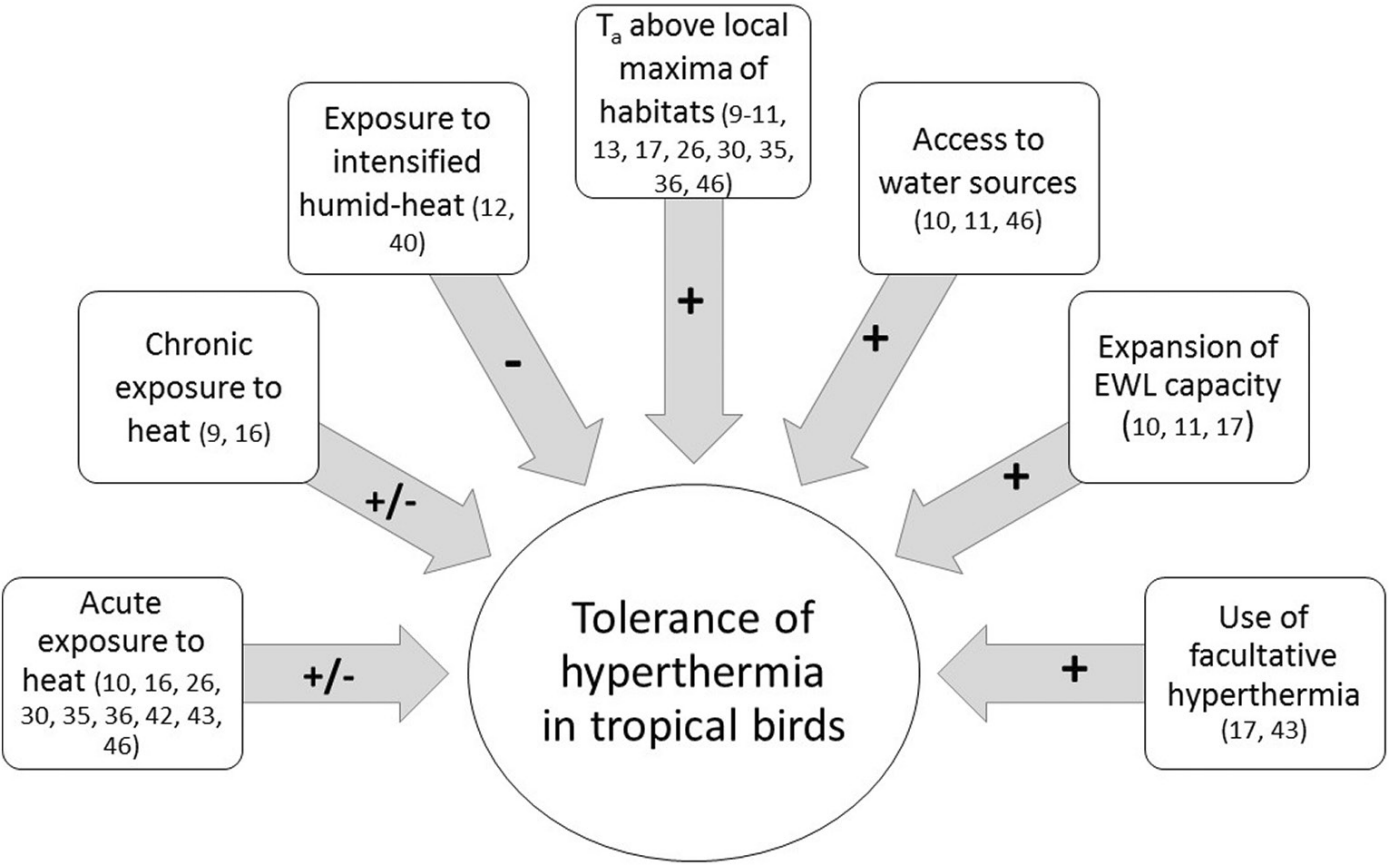
Factors that influence the tolerance of hyperthermia in tropical birds, based on studies in which thermal tolerance was measured through experimental approaches. Reference numbers (in parenthesis) come from the list in the Supplementary Material. Factors had a positive (plus sign), negative (minus sign) or mixed outcome (plus/minus) on birds. **T**_**a**_ = ambient temperature during experiments.

The assumption of a generalized proximity to the limits of thermal tolerance probably stemmed from regarding the T_UC_ as a tolerance threshold (Mitchell et al., 2018). Because the T_UC_ has been considered largely invariable, tropical endotherms would experience thermal stress any time that T_a_ surpasses this limit (Araújo et al., 2013; Huey et al., 2012). Thus, given that current T_a_ lie around the T_UC_ of many tropical birds (Pollock et al., 2021), these are assumed to be constantly exposed to increased costs of thermoregulation or to risks of overheating. We believe that these notions should be reconsidered. First, there is evidence of temporal variation in the T_UC_ in tropical birds, highlighting their potential for acclimatization and adaptation (Pollock et al., 2019). Also, modest hyperthermia is tolerated at T_a_ above the T_UC_ (Table 1), hinting reduced thermoregulatory expenditure. Finally, recent research on temperate birds demonstrates the capacity to adjust the limits of hyperthermia tolerance depending on the prevalent environmental conditions (Freeman et al., 2022). We suggest that an appropriate indicator of whether tropical birds can tolerate T_a_ even higher than the ones currently experienced within their ranges is the response of T_b_ when facing high levels of heat (Figure 2).

Hyperthermia at high T_a_ can be endured by tropical birds for the duration of metabolic experiments—typically 2–4 h—reflecting a flexible response to acute heat exposure (Table 1). Species from hot, xeric regions that have access to water sources tolerate hyperthermia at experimental T_a_ above the maximum of the study areas (~45°C) by increasing EWL rates to dissipate all metabolic and exogenous heat (Dawson & Bennett, 1973; Ehlers & Morton, 1982; Withers & Williams, 1990). Along elevational gradients, highland hummingbirds (Trochilidae) maintain constant T_b_, while mid‐elevation species develop modest hyperthermia, at T_a_ well above the local maxima of their natural habitats (Lasiewski et al., 1967; Schuchmann & Schmidt‐Marloh, 1979a, 1979b; Wolf & Hainsworth, 1972). Tropical birds can also tolerate hyperthermia regularly during the dry season in their natural habitat or during experimental exposure for weeks (Cox, 1961; Nilsson et al., 2016). In addition, one species did not experience hyperthermia when living under experimental worst‐case scenario warming for one year (Thompson et al., 2015). All of this empirical data support the recent discovery that projected warming T_a_ will most likely stay far from eliciting lethal hyperthermia for many tropical birds (Pollock et al., 2021; but see Section 5). Thus, the notion that tropical birds risk thermal stress in their natural habitats because of warming must be reconsidered in the context of their capacity to tolerate elevated T_b_. We note, however, that there is an impending need to explore further their response to chronic heat exposure.

### 3.3 Humidity and thermoregulation in tropical birds

Air moisture is a relevant factor in the avian thermoregulatory response to heat. Humidity along with T_a_ can directly affect T_b_ regulation in birds (Gardner et al., 2016), but their combined effects have not been as exhaustively studied as the impacts of T_a_ alone (i.e., dry heat; Rogers et al., 2021). Humid heat can severely hamper the efficacy of cutaneous evaporative cooling in endotherms at high T_a_ (Buzan & Huber, 2020) when the water vapor pressure in the surrounding air exceeds that of the body surfaces from which water is used to dissipate heat (Boyles et al., 2011). Although dissipating heat through panting might soften the effects of humidity (Gerson et al., 2014), exposure to high humid heat could have a generalized effect across avian taxa of generating more metabolic heat than can be lost through evaporative cooling (van Dyk et al., 2019).

Because the combination of high humidity and high heat is more prevalent in the Tropics than in other climatic zones, tropical birds might frequently resort to dissipating heat convectively via facultative hyperthermia instead (Gardner et al., 2016; van Dyk et al., 2019). In lowland areas with high dew points, facultative hyperthermia may overcome the limitations of the diminished scope for evaporative dissipation of heat loads (Weathers, 1997). This strategy can allow tropical rainforest birds to remain active (e.g., foraging or flying) when exposed to intense sun radiation (Weathers, 1977). Nonevaporative heat dissipation through body structures is an effective thermo‐tolerance mechanism to survive in tropical hot‐humid habitats (Eastick et al., 2019; Tattersall et al., 2009; van de Ven et al., 2016). For instance, the bill plays a key role in the adaptive thermoregulatory response of birds (Tattersall et al., 2017). Notably, the positive association between bill size and humidity appears to be phylogenetically independent and more likely to be determined by environmental conditions (Gardner et al., 2016). For example, larger bills have been measured in individuals of temperate and migratory passerines that live in water‐limited, humid and hot habitats (Greenberg et al., 2012; Luther & Greenberg, 2014).

Despite thriving in mainly hot and humid habitats, knowledge of the response of tropical birds to the joint effects of humidity and heat is still incipient. For example, under constant relative humidity of 45% during experiments, lowland and even highland birds seemed to tolerate the typical T_a_ of the lowland rainforest (Londoño et al., 2017). In contrast, when humidity was not controlled, some lowland passerines quickly became hyperthermic after acute and chronic exposure to moderate T_a_ or failed to survive high T_a_ (Cox, 1961; Prinzinger et al., 1989; Weathers, 1977). Under constant T_a_ of 25°C, EWL in the mountain‐dwelling giant hummingbird (*Patagona gigas*) decreased by ~3‐fold when experimental relative humidity increased from 0% to 90% (Lasiewski et al., 1967). Furthermore, the thermoregulatory advantage of larger bills in tropical birds can fade in highly humid sites that experience extreme maximum temperatures (Gardner et al., 2016). Undoubtedly, more research is needed to improve our understanding of how the combined effects of heat and humidity modify thermal vulnerability in tropical birds.

### 3.4 Microclimate, land‐use change, and thermoregulation

**FIGURE 3:**
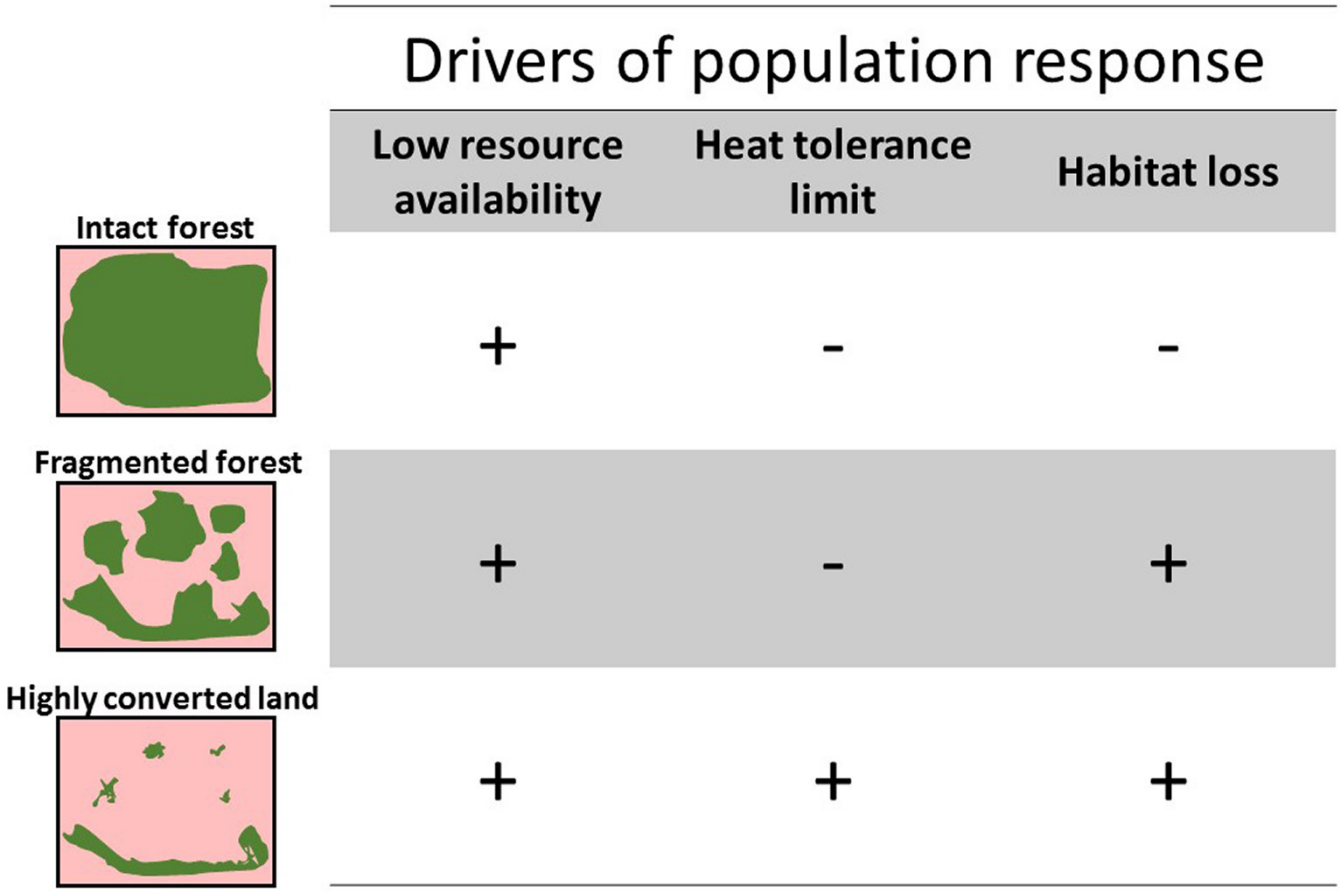
Potential drivers of tropical bird population response to the interactive effects of land use and climate change based on the combination of physiological vulnerability, habitat loss and resource availability. The drawings represent three scenarios of progressive habitat loss, with forest depicted in green and converted lands in light red. Symbols depict our own predictions of how the drivers may affect tropical bird populations, whether strongly (plus) or weakly (minus), in each of the three scenarios.

Tropical birds have long been severely impacted by deforestation, with long‐term abundance declines of terrestrial and understory insectivores of up to 95% in isolated forest fragments (Stouffer et al., 2006). Unfortunately, clearing and degradation of lowland and montane forests continues to spread at alarming rates (Bodart et al., 2013; Ernst et al., 2013; Shearman et al., 2009), even occurring during periods of severe drought (Bullock et al., 2020). Consequently, tropical deforestation can become a major amplifier of climate change. For instance, accumulated local warming in deforested lands now equates to predicted worst‐case scenario warming (Zeppetello et al., 2020). Furthermore, the incidence of heat waves in the Tropics will likely be highest in areas converted to agriculture (Im et al., 2017). The relevant question here is whether this panorama can increase the physiological vulnerability of tropical birds to warming. Based on our synthesis of literature, we believe that the answer may lie at the interplay between the extent of habitat loss, heat tolerance and resource availability (Figure 3).

The loss of natural habitat cover disrupts microclimatic conditions, with potential consequences for biological communities (Guo et al., 2018; Zellweger et al., 2020). For instance, while habitat conversion can reduce total abundance in avian communities, associated warming can alter species‐specific abundances because of differences in heat tolerance (Bowler et al., 2018). Surviving species would not only live under increased average local temperatures but further habitat loss and resource depletion may result in heat waves and drought challenging their thermal limits (Senior et al., 2017). The availability of microclimatic refuges can in fact determine survival for birds when physiological responses, such as panting, become inefficient to deal with extreme heat (Sharpe et al., 2022). This could be particularly true for tropical birds resilient to deforestation in lowland hot and dry habitats (Frishkoff et al., 2016). Likewise, even if montane species can tolerate exposure to increased microsite temperatures under current conditions, they may become vulnerable under progressing warming and habitat loss in the future (Monge et al., 2022).

Under a less extreme scenario, microclimatic buffering provided by even a fragmented forest has the potential to shield birds from increased warming, though less than in intact forest (Ewers & Banks‐Leite, 2013). When facing fragmentation, how likely are tropical birds associated with the forest interior to become physiologically vulnerable to warming? According to the “microclimate hypothesis,” tropical understory birds choose cool, moist and dark microsites within rainforests and changes to these conditions bring physiological vulnerability (Patten & Smith‐Patten, 2012). However, the relative contribution of thermal stress in this vulnerability is still poorly understood. When natural fragmentation occurs, remaining in or around gaps can cause understory birds to become slightly hyperthermic (Jirinec, Rodrigues, et al., 2022). Avoiding large natural gaps would thus be advantageous, considering that these can be <1 ha in area and cover <2% of the entire rainforest tracts (Hunter et al., 2015). This way, birds could escape high humid heat in gaps by staying in close‐canopy sites during the hottest hours of the day. Naturally, the reduction in fragment size would lower the chances of finding microclimatic refugia because the buffering effect drops near forest edges (Ewers & Banks‐Leite, 2013), potentially increasing the vulnerability of understory birds (Patten & Smith‐Patten, 2012; Pollock et al., 2015). Thus, unraveling the degree of thermal stress, if any, of forest‐dependent birds inside tropical rainforests is a pending research avenue. More urgent is to investigate their physiological response during exposure to conditions at the forest edges, where the most drastic microclimatic changes take place.

Finally, abundance declines and community turnover of terrestrial and understory insectivores have been reported inside undisturbed tropical forests. While intuitively linked to climate change, the proximate causes of these rearrangements remain unknown (Blake & Loiselle, 2015; Curtis et al., 2021; Pollock et al., 2022; Stouffer et al., 2021). Negative indirect effects, such as variations in resource availability, offer a plausible explanation (Lister & Garcia, 2018; Neate‐Clegg et al., 2020), especially if those resources are involved in maintaining effective thermoregulation when climatic conditions harshen. Terrestrial insectivorous birds can seasonally track water or prey to fulfill thermoregulatory needs, but increasingly hot and dry conditions might lower habitat quality and increase the birds' vulnerability (Jirinec, Elizondo, et al., 2022). The loss of thermoregulatory resources coupled with the intense climatic conditions may thus drive the disappearance of birds on the interior in intact forests (Curtis et al., 2021). Hopefully, more work would help to unravel if, when and how thermal tolerance is related to these puzzling trends.

## 4 AN UPDATED APPROACH TO ASSESS VULNERABILITY TO WARMING IN TROPICAL BIRDS, KNOWLEDGE GAPS, AND FUTURE RESEARCH DIRECTIONS

**FIGURE 4:**
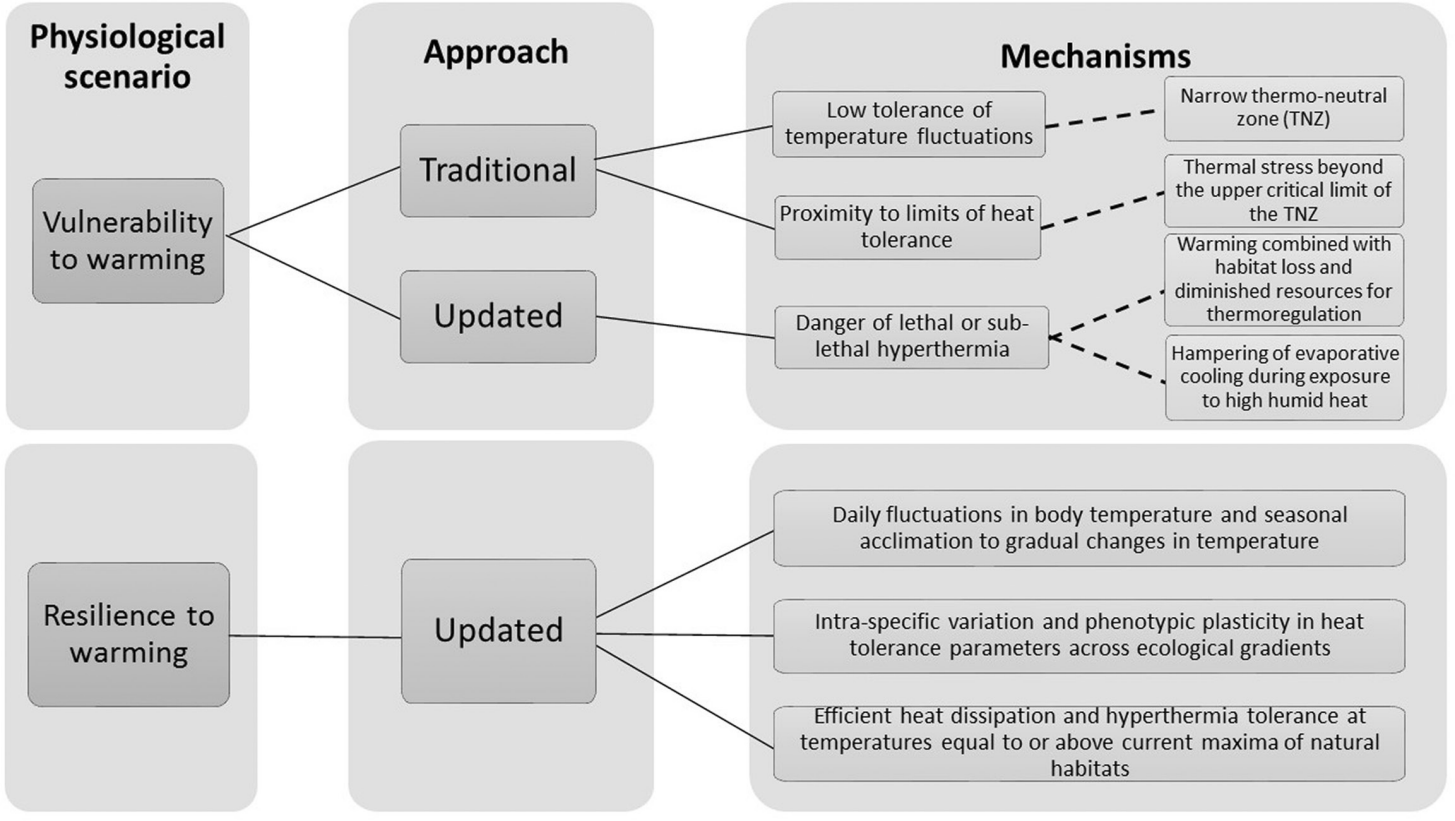
Physiological vulnerability and resilience to warming in tropical birds. The **Traditional Approach** refers to the assumptions commonly used to describe vulnerability to warming in tropical biotas in general whereas the **Updated Approach** is based on our synthesis of literature involving tropical birds. The dashed lines in the **Mechanisms** box connect assumptions with their respective physiological mechanisms in the response to temperature changes.

The empirical data on thermal tolerance allowed us to assess whether the physiological response of tropical birds to heat support the assumption that observed distributional rearrangements are driven by thermal vulnerability. In essence, a narrow thermal tolerance and a proximity to thermal limits do not appear to be prevailing features of tropical birds, not even for high‐elevation species which are alarmingly underrepresented in studies of heat tolerance (Table 1). Therefore, based on the information synthesized, we present an updated approach to re‐assess vulnerability and resilience of tropical birds (Figure 4). The observed distributional rearrangements, from the individual to the community level, seem to result from synergies between land‐use change and microclimatic variation or from indirect effects of climate change on natural habitats and key resources. However, there are still unaddressed topics which limit our knowledge about potential sources of thermal vulnerability (Table 2).

**TABLE 2:** Topics in which knowledge gaps exist, hindering understanding of the physiological response of tropical birds to warming, and suggested lines of research to tackle those gaps.

Topics with knowledge gaps	Suggested lines of research	Example references
EWL and thermal tolerance limits	Move beyond solely analyzing BMR, the TNZ and its limits and assess cooling capacities through the quantification of EWL and maximum tolerable T_a_ and T_b_	Cunningham et al. (2013); Albright et al. (2017); Conradie et al. (2020); Riddell et al. (2019); Freeman et al. (2022)
Long‐term response to sustained levels of warming	Analyze the physiological effects of sustained heat along with survival estimates, changes in body mass, risk of dehydration over consecutive days, limits to cognitive and motor abilities relevant for survival, among others	Thompson et al. (2015); Conradie et al. (2019); Danner et al. (2021)
Thermal tolerance along mountain slopes	Intra‐ and inter‐specific comparisons of heat tolerance limits at different zones along elevational gradients	Soobramoney et al. (2003); Thompson and Downs (2017)
Thermoregulatory consequences of high humid‐heat	Quantify heat dissipation at joint gradients of air humidity and temperature above T_b_ and examine morphological adaptations between populations along ecological gradients to tackle elevated humid heat	Greenberg et al. (2012); Gerson et al. (2014); Luther and Greenberg (2014); Gardner et al. (2016)
Interactive effect of land‐use conversion on microclimates	Measure heat tolerance limits across treatments or gradients of human disturbance along with microclimate variables	Monge et al. (2022)
Physiological response of declining forest‐interior species	Intra‐ and inter‐specific analysis of cooling capacity during acute and chronic exposure to heat at forest interior and edge	

*Note*: The column with example references contain a sample of studies, mostly carried out on birds from extra‐tropical regions, that can help to guide research avenues in the Tropics.

First and foremost, most studies have focused on the BMR and the TNZ. For analyses of vulnerability or resilience to climate change to be complete, a close examination of the abilities for heat dissipation is a requirement. Ideally, more data on the long‐term response should be produced because chronic exposure to heat can impair vital functions over time when birds become unable to dissipate heat efficiently (Conradie et al., 2019). Thus, future studies should consider a careful selection of key parameters to measure vulnerability to warming and how these react to seasonal climatic variation and anomalies. Of such, the EWL and the T_a_ *max* have informed assessments of the probability of extirpation and survival in subtropical birds that inhabit arid zones in which T_a_ variation has intensified as a consequence of climate change (Albright et al., 2017; Riddell et al., 2019). In addition, the variation in the maximum tolerable T_b_ has been analyzed across ecological gradients in subtropical birds (Freeman et al., 2022). Researchers might also analyze the combinations of humidity and T_a_ that severely hamper effective heat dissipation, and are detrimental to survival, to determine species‐specific vulnerability. For instance, changes in rainfall could be particularly problematic to small tropical songbirds given that their reliance on passive heat dissipation could put them at risk during episodes of very high humid heat (Gardner et al., 2016; Gerson et al., 2019).

Additionally, more intra‐ and interspecific studies of species that inhabit environmental gradients (e.g., T_a_, precipitation, aridity) could allow the identification of populations and species more vulnerable to local warming as well as physiological features which could make them more resilient in different parts of their distribution (i.e., phenotypic plasticity) (Cavieres & Sabat, 2008; Tieleman et al., 2002). Birds in general can experience short‐ and long‐term seasonal variation in BMR and EWL (McKechnie et al., 2007; Soobramoney et al., 2003; Thompson & Downs, 2017; Tieleman et al., 2003) but also, and most importantly, cooling capacity and heat tolerance limits can vary in proportion to the severity of variation in environmental conditions, such as T_a_ and humidity (Freeman et al., 2022; Noakes et al., 2016). For instance, some lark species (Alaudidae) responded to increasing aridity along their distributions with lower phylogeny‐independent rates of EWL, suggestive of a plastic response among species (Tieleman et al., 2002). Unfortunately, intraspecific and phenotypic plasticity studies are largely absent for tropical birds. While Puerto Rican todies (*Todus mexicanus*) from a lowland xeric habitat exhibited lower BMR than individuals from montane humid forests, evidence for T_b_ was less definite (Merola‐Zwartjes & Ligon, 2000; Oniki, 1975). Similarly, interspecific studies are scant and show inconsistencies in the patterns of variation. For example, across elevational and ecological gradients, T_b_ and BMR varied in some studies but not in others (Hails, 1983; Londoño et al., 2015, 2017; Seavy, 2006). Given that BMR can only partially explain flexibility in thermal tolerance, more intra‐ and interspecific data on EWL, cooling efficiency, and upper T_b_ limits are urgently needed for species along ecological and elevational gradients.

Finally, we would like to underscore the relevance of considering the human disturbance of natural habitats as the leading cause of vulnerability for birds in the tropical regions (Caro et al., 2022). Tropical birds are well adapted to the abiotic conditions of their natural habitats, but the changes in land use disrupt this balance (Figure 3). In fact, the combination of habitat alterations and climatic variations can drive patterns of extinction and colonization shifts in tropical birds (Beale et al., 2013). Therefore, we believe that the key areas that need to be assessed in order to determine the physiological vulnerability of tropical birds to warming are those directly affected by human activities.

## 5 CONCLUSIONS

Based on our literature review, we propose that many tropical birds are resilient enough to tolerate thermal variation within the range of predicted future levels of warming. Thus, we concur with Pollock et al. (2021) that tropical birds are no more physiologically threatened by warming in the short‐term than birds at other latitudes. Most likely, the global hotspots of imminent avian physiological vulnerability reside in arid regions outside the Tropics [e.g., southern Africa (Conradie et al., 2019), Australia (McKechnie et al., 2012), North American southwest (Albright et al., 2017), and the Iberian Peninsula (Cabello‐Vergel et al., 2022)]. However, this does not mean that tropical birds are physiologically insensitive to warming in general. An increase of 5°C in local average T_a_, as in worst‐case scenarios, could prove challenging to birds from open areas who rely on T_b_–T_a_ gradients for passive heat dissipation, as this strategy would demand higher levels of hyperthermia. An equally vulnerable group are birds from hot, xeric habitats if water sources become absent or reduced during heat waves. Also, the consistent variation in rainfall regimes, which can produce more intense wet and dry seasons (Brawn et al., 2017; Chadwick et al., 2016), has the potential to alter the frequency of stronger humid‐heat events and extend the length of the dry season. Climate change is the world's greatest concern at the scientific and public‐opinion level but this has taken the focus away from other, more imminent, threats to biodiversity such as anthropogenic habitat loss and degradation (Caro et al., 2022). Land‐use change does not only affect tropical bird diversity directly but also reinforces climate‐driven threats by altering the microclimate birds are exposed to (Monge et al., 2022). Therefore, adaptive measures such as protecting vast areas covered by forest (Stouffer et al., 2011), especially along ecological gradients (Brodie et al., 2012) or, alternatively, improving land management strategies (Oliver & Morecroft, 2014) are the most promising approaches to safeguard the diversity of tropical birds.

## AUTHOR CONTRIBUTIONS

**Otto Monge:** Conceptualization (equal); data curation (lead); formal analysis (lead); funding acquisition (equal); writing – original draft (lead). **Ivan Maggini:** Conceptualization (equal); writing – review and editing (equal). **Christian H. Schulze:** Funding acquisition (equal); supervision (equal); writing – review and editing (equal). **Stefan Dullinger:** Funding acquisition (equal); supervision (lead); writing – review and editing (equal). **Leonida Fusani:** Funding acquisition (equal); supervision (lead); writing – review and editing (equal).

## CONFLICT OF INTEREST STATEMENT

None declared.

## Supporting information


Data S1.

## Data Availability

No datasets were generated for this manuscript and all data used is presented within the manuscript as well as in the Online Supplementary Material.
