# Supplementary material for: Physiologically vulnerable or resilient? Tropical birds, global warming, and redistributions
Source: Ecol Evol. 2023 Apr 18;13(4):e9985. doi: 10.1002/ece3.9985 (PMC10111238; doi:10.1002/ece3.9985)
Supplement: Supplementary file 1 — Data S1. [file ECE3-13-e9985-s001.docx]

Supplementary material

**Physiologically vulnerable or resilient? Tropical birds, global warming and redistributions**

Otto Monge^*^, Ivan Maggini, Christian H. Schulze, Stefan Dullinger, Leonida Fusani

^*^Correspondence

Otto Monge

monge_otto@hotmail.com

Contents

1. Materials and methods

2. List of bibliographical references from the literature search used in our synthesis

3. Supplementary Table 1.

1. **Materials and methods**

The search for scientific articles was made using keywords in the ISI Web of Science (WoS) and Google Scholar (GS) databases. We did not set a specific time range for the date of publication of papers, in order to capture old as well as recent information on the field of tropical bird thermal physiology. For the search in WoS, the keywords were entered in two groups and then the groups were merged in a new search. With the first group of keywords: 1- *TS=(“thermal tolerance” OR “thermal stress” OR “thermal physiology” OR “thermoregulat*” OR “metabolic rate”) AND TS=(tropic* OR Tropic*) AND TS=(bird* OR avian OR avifauna)*, we aimed to look for any published studies in which the thermal tolerance of tropical birds was assessed. To this, the following group of keywords was added: 2- *TS=(“global warming” OR “climate change” OR “climate warming” OR “warming temperature”)*. Additional articles were retrieved by entering the searching criteria “*thermal tolerance tropical bird climate change*” and “*tropical bird ecophysiology climate change*” into the GS search engine. Before filtering the retrieved papers, duplicates from both searches were identified and excluded. Literature on the topics of the effects of humidity and micro-climate variation on tropical birds was searched using keywords on the GS database. For humidity, we used “*tropical bird humidity warming*” and for micro-climate variations we used “*microclimate tropical bird physiology*”. Finally, we also examined the reference lists of retrieved articles to look for papers not found in our search.

The resulting list of articles had their titles and abstracts screened to detect and exclude works that took place outside the world’s tropics or that were not broadly related to climate change or thermal physiology. A full-text filtering using selection criteria followed to include only studies that were carried out on native wild-caught tropical bird species, leaving out domesticated, semi-domesticated, or captive-raised birds. Search criteria also filtered out studies that measured metabolic parameters not related to thermal tolerance. Literature reviews and papers in which the role of tropical birds could not be precisely identified (e.g. broad global meta-analyses of vertebrates) were not included in the synthesis.

1. **List of bibliographical references from the literature search used in our synthesis**
2. Bartholomew GA, Vleck CM, Bucher TL. 1983. Energy metabolism and nocturnal hypothermia in two tropical passerine frugivores, *Manacus vitellinus* and *Pipra mentalis*. Physiol. Zool. 56:370–379. doi:10.1086/physzool.56.3.30152601.
3. Bech C, Abe AS, Steffensen JF, Berger M, Bicudo JEPW. 1997. Torpor in three species of Brazilian hummingbirds under semi-natural conditions. Condor 99:780–788. doi:10.2307/1370489.
4. Berger M, Hart JS. 1972. Die Atmung beim Kolibri *Amazilia fimbriata* während des Schwirrfluges bei verschiedenen Umgebungstemperaturen. J. Comp. Physiol. B 81:363–380. doi:10.1007/BF00697756.
5. Bosque C, Pacheco MA, Siegel RB. 1999. Maintenance energy costs of two partially folivorous tropical passerines. Auk 116:246–252. doi:10.2307/4089474.
6. Bucher TL. 1981. Oxygen consumption, ventilation and respiratory heat loss in a parrot, *Bolborhynchus lineola*, in relation to ambient temperature. J. Comp. Physiol. B 142:479–488. doi:10.1007/BF00688979.
7. Bucher TL, Worthington A. 1982. Nocturnal hypothermia and oxygen consumption in manakins. Condor 84:327–331. doi:10.2307/1367377.
8. Carpenter FL. 1974. Torpor in an Andean hummingbird: its ecological significance. Science 183:545–547. doi:10.1126/science.183.4124.545.
9. Cheke RA. 1970. Temperature rhythms in African montane sunbirds. Ibis 113:500–506. doi:10.1111/j.1474-919X.1971.tb05184.x.
10. Cox GW. 1961. The relation of energy requirements of tropical finches to distribution and migration. Ecology 42:253–266. doi:10.2307/1932077.
11. Dawson WR, Bennett AF. 1973. Roles of metabolic level and temperature regulation in the adjustment of western plumed pigeons (*Lophophaps ferruginea*) to desert conditions. Comp. Biochem. Physiol. A 44:249–266. doi:10.1016/0300-9629(73)90478-7.
12. Ehlers R, Morton ML. 1982. Metabolic rate and evaporative water loss in the least seed-snipe, *Thinocorus rumicivorus*. Comp. Biochem. Physiol. A 73:233–235. doi:10.1016/0300-9629(82)90061-5.
13. Gardner JL, Symonds MRE, Joseph L, Ikin K, Stein J, Kruuk LEB. 2016. Spatial variation in avian bill size is associated with humidity in summer among Australian passerines. Clim. Change Responses 3:3–11. doi:10.1186/s40665-016-0026-z.
14. Hainsworth FR, Wolf LL. 1970. Regulation of oxygen consumption and body temperature during torpor in a hummingbird, *Eulampis jugularis*. Science 168:368–369. doi:10.1126/science.168.3929.368.
15. Krüger K, Prinzinger R, Schuchmann K-L. 1982. Torpor and metabolism in hummingbirds. Comp. Biochem. Physiol. A 73:679–689. doi:10.1016/0300-9629(82)90275-4.
16. Lasiewski RC, Hubbard S, Moberly W. 1964. Energetic relationships of a very small passerine bird. Condor. 66:212–220. doi:10.2307/1365646.
17. Lasiewski RC, Weathers WW, Bernstein MH. 1967. Physiological responses of the giant hummingbird, *Patagona gigas*. Comp. Biochem. Physiol. A 23:797–813. doi:10.1016/0010-406X(67)90342-8.
18. Lasiewski RC, Dawson WR, Bartholomew GA. 1970. Temperature regulation in the little Papuan frogmouth, *Podargus ocellatus*. Condor 72:332–338. doi:10.2307/1366012.
19. Londoño GA, Chappell MA, Jankowski JE, Robinson SK. 2017. Do thermoregulatory costs limit altitude distributions of Andean forest birds? Funct. Ecol. 31:204–215. doi:10.1111/1365-2435.12697.
20. MacMillen RE. 1981. Nonconformance of standard metabolic rate with body mass in Hawaiian Honeycreepers. Oecologia 49:340–343. doi:10.1007/BF00347595.
21. Marschall U, Prinzinger R. 1991. Vergleichende Ökophysiologie von fünf Prachtfinkenarten (Estrildidae). J. Ornithol. 132:319–323. doi:10.1007/BF01640540.
22. Mata A. 2010. Metabolic rate and specific dynamic action of the Red-legged Honeycreeper, a nectar-feeding Neotropical passerine. Comp. Biochem. Physiol. A 157:291–296. doi:10.1016/j.cbpa.2010.07.018.
23. McNab BK. 2013. The ecological energetics of birds in New Guinea. Bull. Florida Mus. Nat. Hist. 52:95–159.
24. McNab BK, Bonaccorso FJ. 1994. The energetics of Australasian swifts, frogmouths, and nightjars. Physiol. Zool. 68:245–261. doi:10.1086/physzool.68.2.30166502.
25. Merola-Zwartjes M. 1998. Metabolic rate, temperature regulation, and the energetic implications of roost nests in the bananaquit (*Coereba flaveola*). Auk 15:780–786. doi:10.2307/4089429.
26. Merola-Zwartjes M, Ligon J. 2000. Ecological energetics of the Puerto Rican tody: Heterothermy, torpor, and intra-island variation. Ecology 81:990–1003. doi:10.1890/0012-9658(2000)081[0990:EEOTPR]2.0.CO;2.
27. Monge O, Schulze CH, Dullinger S, Fusani L, Maggini I. 2022. Unshaded coffee imposes a heavier load on thermoregulation than shaded coffee for birds in a tropical mountainous region. Glob. Ecol. Conserv. 36:e02117. doi:10.1016/j.gecco.2022.e02117.
28. Morrison P. 1962. Modification of body temperature by activity in Brazilian hummingbirds. Condor 64:315–323. doi:10.2307/1365371.
29. Pearson O. 1953. Use of caves by hummingbirds and other species at high altitudes in Peru. Condor 55:17–20. doi:10.2307/1364918.
30. Pollock HS, Brawn JD, Agin TJ, Cheviron ZA. 2019. Differences between temperate and tropical birds in seasonal acclimatization of thermoregulatory traits. J. Avian Biol. 50. doi:10.1111/jav.02067.
31. Pollock HS, Brawn JD, Cheviron ZA. 2021. Heat tolerances of temperate and tropical birds and their implications for susceptibility to climate warming. Funct. Ecol. 35:93–104. doi:10.1111/1365-2435.13693.
32. Prinzinger R. 1988. Energy metabolism, body-temperature and breathing parameters in nontorpid blue-naped Mousebirds *Urocolius macrourus*. J. Comp. Physiol. B 157:801–806. doi:10.1007/BF00691011.
33. Prinzinger R, Lübben I, Schuchmann K-L. 1989. Energy metabolism and body temperature in 13 sunbird species (Nectariniidae). Comp. Biochem. Physiol. A 92:393–402. doi:10.1016/0300-9629(89)90581-1.
34. Ruschi A. 1949. Observações sobre Trochilideos. Bol. Mus. Biol. Mello Leitão 1.
35. Scholander P, Hock R, Walters V, Johnson F, Irving L. 1950. Heat regulation in some arctic and tropical mammals and birds. Biol. Bull. 99:237–258. doi:10.2307/1538741.
36. Schuchmann K-L, Schmidt-Marloh D. 1979a. Temperature regulation in non-torpid hummingbirds. Ibis 121:354–356. doi:10.1111/j.1474-919X.1979.tb06858.x.
37. Schuchmann K-L, Schmidt-Marloh D. 1979b. Metabolic and thermal responses to heat and cold in streamertail hummingbirds (*Trochilus polytmus* and *Trochilus scitulus*, Trochilidae). Biotropica 11:123–126. doi:10.2307/2387787.
38. Seavy NE. 2006. Physiological correlates of habitat association in East African sunbirds (Nectariniidae). J. Zool. 270:290–297. doi:10.1111/j.1469-7998.2006.00138.x.
39. Seavy NE, McNab BK. 2007. Energetics of East African pycnonotids. Biotropica 39:114–119. doi:10.1111/j.1744-7429.2006.00216.x.
40. Steiger SS, Kelley JP, Cochran WW, Wikelski M. 2009. Low metabolism and inactive lifestyle of a tropical rain forest bird investigated via heart-rate telemetry. Physiol. Biochem. Zool. 82: 580–589. doi:10.1086/605336.
41. van Dyk M, Noakes MJ, McKechnie AE. 2019. Interactions between humidity and evaporative heat dissipation in a passerine bird. J. Comp. Physiol. B. 189:299–308. doi:10.1007/s00360-019-01210-2.
42. Warren JW. 1960. Temperature fluctuation in the Smooth-billed Ani. Condor 62:293–298. doi:10.2307/1365519.
43. Weathers W. 1977. Temperature regulation in the dusky munia, *Lonchura fuscans* (Cassin) (Estrildidae). Australian J. Zool. 25:193–199. doi:10.1071/ZO9770193.
44. Weathers W. 1997. Energetics and thermoregulation by small passerines of the humid, lowland tropics. Auk 114:341–353. doi:10.2307/4089237.
45. Weathers W, van Riper C. 1982. Temperature regulation in two endangered Hawaiian honeycreepers: The palila (*Psittirostra bailleui*) and the Laysan finch (*Psittirostra cantans*). Auk 99:667–674. doi:10.1093/auk/99.4.667.
46. Wikelski M, Spinney L, Schelsky W, Scheuerlein A, Gwinner E. 2003. Slow pace of life in tropical sedentary birds: A common-garden experiment on four stonechat populations from different latitudes. Proc. R. Soc. B 270:2383–2388. doi:10.1098/rspb.2003.2500.
47. Withers PC, Williams JB. 1990. Metabolic and respiratory physiology of an arid-adapted Australian bird, the spinifex pigeon. Condor 92:961–969. doi:10.2307/1368732.
48. Witt HH, Schuchmann K-L, Sutton R. 1981. Zur biologie des jamaikanischen Grüntodi *Todus todus*. Bonn Zool. Beitr. 32:103–110.
49. Wolf LL, Hainsworth FR. 1972. Environmental influence on regulated body temperature in torpid hummingbirds. Comp. Biochem. Physiol. A 41:167–173. doi:10.1016/0300-9629(72)90044-8.
50. Yarbrough CG. 1971. The influence of distribution and ecology on the thermoregulation of small birds. Comp. Biochem. Physiol. 39:235–266. doi:10.1016/0300-9629(71)90082-X.

**Supplementary Table 1.** Ambient temperatures (T_a_) relevant to the physiological tolerance of cold (blue columns) and heat (red columns) in tropical birds measured in studies retrieved from our literature search (n=47). Included are the flow rates of air incoming to the chamber in metabolic experiments and the method employed to measure body temperature (T_b_); when not specified: n.s. In some studies, neither metabolic rates nor T_b_ were measured: n.m. **T_LC_** and **T_UC_** = lower and upper critical T_a_ of the thermo-neutral zone (TNZ), **T_hypo_** and **T_hyper_** = T_a_ in which hypo- and hyperthermia develop; **T_ther_** = T_a_ that triggers a thermogenesis response to cold (i.e. metabolic heat production); **T_EWL_** = T_a_ that forces a sharp increase in the rate of evaporative water loss (EWL); **T_a_*min*** and **T_a_*max*** = minimum and maximum tolerable T_a_.

| Reference | Species (family) | Flow rate (ml/min)^*^ | T_b_ method^†^ | T_a_*min* | T_ther_ | T_hypo_ | T_LC_ | T_UC_ | T_hyper_ | T_EWL_ | T_a_*max* |
| --- | --- | --- | --- | --- | --- | --- | --- | --- | --- | --- | --- |
| Bartholomew et al. (1983) | 2 manakin species (Pipridae) | 150 | Thermocouple in cloaca |  | X | X | X |  |  |  |  |
| Bech et al. (1997) | 3 hummingbird species (Trochilidae) | n.m. | Thermocouple subcutaneous (under wing) |  | X |  |  |  |  |  |  |
| Berger and Hart (1972) | *Amazilia fimbriata* (Trochilidae) | 2000 | Thermocouple in cloaca |  |  |  |  |  |  | X |  |
| Bosque et al. (1999) | 2 saltator species (Thraupidae) | 658–762 | Thermocouple in cloaca |  |  | X | X |  |  |  |  |
| Bucher (1981) | *Bolborhynchus lineola* (Psittacidae) | 400–600 | Thermocouple in cloaca |  |  |  | X | X |  | X |  |
| Bucher and Worthington (1982) | 2 manakin species (Pipridae) | 90–215 | Thermocouple in cloaca |  | X |  |  |  |  |  |  |
| Carpenter (1974) | *Oreotrochilus estella* (Trochilidae) | n.m. | Analog thermometer in cloaca |  | X | X |  |  |  |  |  |
| Cheke (1970) | 4 sunbird species (Nectariniidae) | n.m. | Thermistor probe in cloaca |  | X |  |  |  |  |  |  |
| Cox (1961) | 4 species from various families | n.m. | n.m. | X |  |  |  |  |  |  | X |
| Dawson and Bennett (1973) | *Geophaps plumifera* (Columbidae) | 330–3300 | Analog thermometer in cloaca |  |  |  | X | X | X | X | X |
| Ehlers & Morton (1982) | *Thinocorus rumicivorus* (Thinocoridae) | 800–2200 | Thermistor probe in cloaca |  |  |  | X | X |  | X |  |
| Hainsworth and Wolf (1970) | *Eulampis jugularis* (Trochilidae) | 150–550 | Thermocouple in cloaca |  | X | X | X |  |  |  |  |
| Krüger et al. (1982) | 18 hummingbird species (Trochilidae) | 333 | Digital thermometer under wing (axilla) |  | X | X |  |  |  |  |  |
| Lasiewski et al. (1964) | *Estrilda troglodytes* (Estrildidae) | 122–152 | n.m. |  |  |  | X |  |  | X |  |
| Lasiewski et al. (1967) | *Patagona gigas* (Trochilidae) | 950–2500 | Thermocouple intra-muscular (pectoral) |  |  | X | X |  |  |  |  |
| Lasiewski et al. (1970) | *Podargus ocellatus* (Podargidae) | 300–3000 | Thermocouple in cloaca |  |  |  | X | X | X | X |  |
| Londoño et al. (2017) | 215 species from various families | 200–1200 | Thermocouple in cloaca |  |  |  | X |  |  |  |  |
| MacMillen (1981) | 3 Hawaiian honeycreeper species (Fringillidae) | 800 | n.m. |  |  |  | X |  |  |  |  |
| Marschall & Prinzinger (1991) | 5 finch species (Estrildidae) | 667 | Digital thermometer in cloaca |  |  |  | X | X |  |  |  |
| Mata (2010) | *Cyanerpes cyaneus* (Thraupidae) | 200–300 | Thermocouple in cloaca |  |  |  | X | X |  |  |  |
| McNab (2013) | 79 species from various families | n.s. | Thermocouple in cloaca |  |  | X | X | X | X |  |  |
| McNab & Bonaccorso (1994) | 6 species from various families | 90–2200 | Thermocouple in cloaca |  |  | X | X | X | X |  |  |
| Merola-Zwartjes (1998) | *Coereba flaveola* (Coerebidae) | 150 | Thermocouple in cloaca |  |  | X | X | X | X |  |  |
| Merola-Zwartjes & Ligon (2000) | *Todus mexicanus* (Todidae) | 145–155 | Thermocouple in cloaca |  |  | X | X | X | X |  |  |
| Morrison (1962) | 3 hummingbird species (Trochilidae) | n.m. | Thermistor probe under wing (axilla) |  | X | X |  |  |  |  |  |
| Monge et al. (2022) | 4 species from various families | 1000 | PIT tag subcutaneous (intra-scapulae) |  |  |  |  |  |  |  |  |
| Pearson (1953) | *Oreotrochilus estella* (Trochilidae) | n.m. | n.s. method in cloaca or esophagus |  |  | X |  |  |  |  |  |
| Pollock et al. (2019) | 41 species from various families | 300–1500 | n.m. |  |  |  | X | X |  |  |  |
| Pollock et al. (2021) | 58 species from various families | 500–3000 | PIT tag in cloaca |  |  |  |  | X | X |  |  |
| Prinzinger (1988) | *Urocolius macrourus* (Coliidae) | 667 | Digital thermometer in cloaca |  |  | X | X |  |  |  |  |
| Prinzinger et al. (1989) | 13 sunbird species (Nectariniidae) | 500 | Digital thermometer in cloaca |  |  | X | X | X | X |  |  |
| Ruschi (1949) | 9 hummingbird species (Trochilidae) | n.m. | n.s. method in cloaca |  |  | X |  |  |  |  |  |
| Scholander et al. (1950) | 2 species from various families | n.s. | n.m. |  |  |  | X |  |  |  |  |
| Schuchmann & Schmidt-Marloh (1979a) | 2 hummingbird species (Trochilidae) | 833 | Thermocouple under wing (axilla) |  |  | X |  |  | X |  |  |
| Schuchmann & Schmidt-Marloh (1979b) | 2 hummingbird species (Trochilidae) | 833 | Thermocouple under wing (axilla) |  |  | X | X | X | X |  |  |
| Seavy (2006) | 7 sunbird species (Nectariniidae) | 375–400 | Thermocouple in cloaca |  |  |  | X |  | X |  |  |
| Seavy & McNab (2007) | 3 bulbul species (Pycnonotidae) | 375–400 | Thermocouple in cloaca |  |  |  | X |  |  |  |  |
| Steiger et al. (2009) | *Hylophylax naevioides* (Thamnophilidae) | 1000 | Thermocouple in cloaca |  |  | X | X | X | X |  |  |
| Warren et al. (1960) | *Crotophaga ani* (Cuculidae) | n.m. | Analog thermometer in cloaca |  |  | X |  |  |  |  |  |
| Weathers (1977) | *Lonchura fuscans* (Estrildidae) | 250–600 | Analog thermometer in cloaca |  |  |  | X | X | X | X | X |
| Weathers (1997) | *Sporophila corvina* (Thraupidae) | 300–670 | Thermistor probe in cloaca |  |  |  | X | X | X | X |  |
| Weathers & van Riper (1982) | 2 Hawaiian honeycreeper species (Fringillidae) | 470–600 | Thermistor probe in cloaca |  |  |  | X | X | X | X |  |
| Wikelski et al. (2003) | *Saxicola torquatus* (Muscicapidae) | 900 | n.m. |  |  |  | X |  |  |  |  |
| Withers and Williams (1990) | *Geophaps plumifera* (Columbidae) | 1200 | Thermocouple in cloaca |  |  |  | X | X | X | X |  |
| Witt et al. (1981) | *Todus todus* (Todidae) | n.m. | Thermistor probe in cloaca |  |  | X |  |  |  |  |  |
| Wolf & Hainsworth (1972) | 2 hummingbird species (Trochilidae) | 150–500 | Thermocouple in cloaca |  | X | X | X |  |  |  |  |
| Yarbrough (1971) | *Trogon rufus* (Trogonidae) | n.s. | Thermistor probe in proventriculus |  |  |  | X |  |  |  |  |
|  | Total |  |  | 1 | 9 | 21 | 32 | 19 | 17 | 10 | 3 |
|  |  |  |  | 63 | | | | 49 | | | |

^*^ Flow rates were adjusted over a wide range of values in studies, according to the specific context of each one. It should be noted that lower flow rates would mirror air conditions from tropical areas (i.e. higher humidity, even if not quantified) more accurately than high rates. In this context, the response of birds during trials under low flow rate conditions could faithfully represent their response in their natural habitats, adding value to understand their vulnerability to warming.

^†^Cloacal measurements have been commonly used in avian thermoregulation studies (reviewed by McCafferty et al. 2015. Animal Biotelemetry, 33. 10.1186/s40317-015-0075-2). Although studies comparing the outputs of measuring cloacal T_b_ against other approaches (e.g. skin, intra-scapular or intra-peritoneal T_b_) are very rare, at T_a_ approaching 20ºC the difference between T_b_ measured using thermocouples inserted in the cloaca versus T_b_ using PIT tags inserted between the scapulae in blue tits (*Cyanistes caeruleus*, Paridae) was lower than 1ºC (Andreasson et al. 2020. J Comp Physiol B 190:349–359. doi:10.1007/s00360-020-01266-5).
